# The significance of c.690G>T polymorphism (rs34529039) and expression of the *CEBPA* gene in ovarian cancer outcome

**DOI:** 10.18632/oncotarget.11822

**Published:** 2016-09-02

**Authors:** Bozena Konopka, Lukasz Michal Szafron, Ewa Kwiatkowska, Agnieszka Podgorska, Aleksandra Zolocinska, Barbara Pienkowska-Grela, Agnieszka Dansonka-Mieszkowska, Anna Balcerak, Martyna Lukasik, Anna Stachurska, Agnieszka Timorek, Beata Spiewankiewicz, Mona El-Bahrawy, Jolanta Kupryjanczyk

**Affiliations:** ^1^ Department of Pathology and Laboratory Diagnostics, Maria Sklodowska-Curie Memorial Cancer Center and Institute of Oncology, Warsaw, Poland; ^2^ Department of Genetics, Maria Sklodowska-Curie Memorial Cancer Center and Institute of Oncology, Warsaw, Poland; ^3^ Department of Molecular and Translational Oncology, Maria Sklodowska-Curie Memorial Cancer Center and Institute of Oncology, Warsaw, Poland; ^4^ Department of Applied Pharmacy and Bioengineering, Medical University of Warsaw, Warsaw, Poland; ^5^ Department of Obstetrics, Gynecology and Oncology, 2nd Faculty of Medicine, Medical University of Warsaw and Brodnowski Hospital, Warsaw, Poland; ^6^ Department of Gynecologic Oncology, Maria Sklodowska-Curie Memorial Cancer Center and Institute of Oncology, Warsaw, Poland; ^7^ Department of Histopathology, Imperial College London, UK

**Keywords:** ovarian cancer, CEBPA, synonymous polymorphism, gene expression, DNA-damaging chemotherapy

## Abstract

The *CEBPA* gene is known to be mutated or abnormally expressed in several cancers. This is the first study assessing the clinical impact of *CEBPA* gene status and expression on the ovarian cancer outcome. The *CEBPA* gene sequence was analyzed in 118 ovarian cancer patients (44 platinum/cyclophosphamide (PC)-treated and 74 taxane/platinum (TP)-treated), both in tumors and blood samples, and in blood from 236 healthy women, using PCR-Sanger sequencing and Real-Time quantitative PCR (qPCR)-based genotyping methods, respectively. The *CEBPA* mRNA level was examined with Reverse Transcription quantitative PCR (RT-qPCR). The results were correlated to different clinicopathological parameters. Thirty of 118 (25.4%) tumors harbored the *CEBPA* synonymous c.690G>T polymorphism (rs34529039), that we showed to be related to up-regulation of *CEBPA* mRNA levels (p=0.0059). The presence of the polymorphism was significantly associated with poor prognosis (p=0.005) and poor response to the PC chemotherapy regimen (p=0.024). In accordance, elevated *CEBPA* mRNA levels negatively affected patient survival (p<0.001) and tumor response to the PC therapy (p=0.014). The rs34529039 SNP did not affect the risk of developing ovarian cancer. This is the first study providing evidence that the c.690G>T, p.(Thr230Thr) (rs34529039) polymorphism of the *CEBPA* gene, together with up-regulation of its mRNA expression, are negative factors worsening ovarian cancer outcome. Their adverse clinical effect depends on a therapeutic regimen used, which might make them potential prognostic and predictive biomarkers for response to DNA-damaging chemotherapy.

## INTRODUCTION

Ovarian cancer is the most lethal gynecological malignancy and it remains the fifth most common cause of cancer-related death in women [1]. Surgical resection followed by the treatment with platinum compounds and taxanes is currently the standard therapy for patients with this disease. The majority of ovarian cancer patients develop resistance to chemotherapy and relapse. Consequently, the 5-years overall survival of patients with advanced ovarian carcinoma is merely 30% [2]. Efforts to improve patient survival include a search for genetic risk factors, prognostic biomarkers and biomarkers predictive of response to chemotherapy.

CCAAT enhancer-binding protein alpha (CEBPA) is a member of the bZIP family of transcription factors, encoded by an intronless gene localized in chromosome 19q13.1 [3]. CEBPA is expressed in highly differentiated tissues; it promotes cell differentiation by transcriptional up-regulation of lineage-specific genes [4]. It also inhibits cell proliferation at the G1 phase of the cell cycle by interacting with other proteins [5, 6], such as: p21, CDK2, CDK4 and E2F, or by regulating the SWI/SNF chromatin-remodeling complex. CEBPA takes part in the regulation of hematopoiesis [7, 8], and terminal differentiation of many cell types, including adipocytes, hepatocytes, and different epithelial cells [4, 9].

To date, there are no data in the literature showing how alterations in the *CEBPA* gene sequence and its expression may affect ovarian cancer prognosis and tumor response to chemotherapy. In our earlier pilot study, we identified this gene as a negative prognostic factor in ovarian cancer patients treated with DNA-damaging agents [8]. Here, we aimed to evaluate the prevalence of *CEBPA* gene mutations and polymorphisms in ovarian cancer patients, and also to look for associations between genetic alterations found and changes in the *CEBPA* mRNA expression levels.

## RESULTS

### Genotyping studies

We identified four types of known *CEBPA* polymorphisms: three synonymous single nucleotide polymorphisms (SNPs) and one duplication (Table 1). The most frequent alteration was: c.690G>T, p.(Thr230Thr), i.e., the SNP no. rs34529039, which was detected in 30 of 118 (25.4%) ovarian cancer patients (both in tumors and blood samples). This polymorphism seemed not to influence the risk of developing ovarian cancer, since its frequency in healthy individuals 47/236 (19.9%) was similar to that observed in tumors (logistic regression model adjusted for age p=0.204). The second SNP (rs752254340), i.e., c.402G>A, p.(Ala134Ala) was present in four of 118 patients (3.4%), while the third SNP (rs192240793), i.e., c.573C>T, p.(His191His) – in one of 118 patients (0.8%). We did not assess the frequency of the last two SNPs in healthy individuals due to their rare occurrence in tumors, and the low probability of heterozygosity in the default global population, equaling 0.1% and 3.3%, respectively [10]. Additionally, nine of 118 tumors (7.6%) had an in-frame duplication of six nucleotides c.584_589dupACCCGC, p.(His195_Pro196dup) in the histidine and proline rich region (HPR) of the CEBPA TAD2 domain. This alteration was also present with a similar frequency (10/127; 7.9%) in healthy controls. No pathogenic *CEBPA* gene mutations were found.

**Table 1:** A detailed numerical characteristics of groups used in gene expression and genotyping studies along with the results of genotyping

Group	Type of expression analysis	Genotyped samples	c.690G>T carriers	c.584_589dupACCCGC carriers	c.402G>A carriers	c.573C>T carriers
**PC-treated patients**	RT-qPCR	32	8	2	2	0
	none	12	3	0	0	0
** Partial sum**		44	11	2	2	0
**TP-treated patients**	RT-qPCR	74	19	7	2	1
** All patients**		**118**	**30 (1)**	**9 (0)**	**4 (1)**	**1 (0)**
**Healthy controls**	NA	236	47 (1)^a^	10 (0)^b^	NA^c^	NA^c^
** Total sum**		**354**	**77 (2)**	**19 (0)**	**4 (1)**	**1 (0)**

NA – not applicable; Values in brackets represent the number of homozygotes for each polymorphism;

a The polymorphism was assessed in the entire group of 236 healthy women using the qPCR-based method;

b The polymorphism was assessed in a subgroup of 127 healthy women using the PCR and Sanger sequencing methods;

c The polymorphism was not assessed in healthy individuals.

### The *CEBPA* gene alterations and clinical endpoints

**Figure 1:**
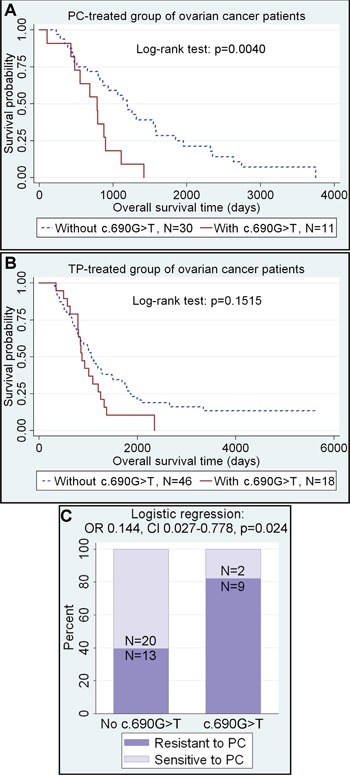
The c.690G>T SNP in the *CEBPA* gene as a negative prognostic and predictive factor **A-B.** The presence of c.690G>T SNP increases the risk of death, but only in ovarian cancer patients treated with PC. Numbers of specimens below the Kaplan-Meier plots refer to completed observations only. **C.** The same polymorphism significantly increases the resistance of tumors to DNA-damaging agents.

We evaluated the clinical significance of the two most common *CEBPA* gene polymorphisms identified herein, c.690G>T, p.(Thr230Thr) and c.584_589dupACCCGC, p.(His195_Pro196dup), in ovarian cancer patients treated with platinum/cyclophosphamide (PC) or taxane/platinum (TP) compounds. Clinical associations were found only for the c.690G>T, p.(Thr230Thr) SNP (Table 2). Multivariate statistical analyses revealed a positive association between the presence of this polymorphism and poor clinical outcome in all patients (the joined PC- and TP-treated group) (HR 1.771, 95% CI 1.129-2.777, p=0.013). However, the analysis in the subgroups of patients treated with either the PC or TP revealed that the *CEBPA* c.690 G>T SNP was associated with more than 3-fold increase of the risk of disease-related death in the PC-treated patients (HR 3.287, 95% CI 1.443-7.490, p=0.005). Noteworthy, such a correlation was not found in the TP-treated group, despite its larger size (Table 2, Figure 1A-1B).

**Table 2:** The c.690G>T polymorphism of the *CEBPA* gene – statistical results of the multivariate analysis of prognosis (Cox proportional hazards model) and prediction (logistic regression model) in the PC-treated group, TP-treated group, and the joined PC- and TP-treated groups of ovarian cancer patients

PC regimen				
Variable name	Analysis of prognosis		Analysis of prediction	
	OS (41/44)^a^ HR [95% CI] p	DFS (27/29)^a^ HR [95% CI] p	PS (22/44)^a^ OR [95% CI] p	CR (29/44)^a^ OR [95% CI] p
**c.690G>T present vs absent**	**3.287 [1.443-7.490] 0.005**	NS	**0.144 [0.027-0.778] 0.024**	0.188 [0.033-1.063] 0.059
**Type (serous vs non-serous)**	0.347 [0.138-0.873] 0.025		-	35.37 [2.395-522.2] 0.009
**Rt ≤ 2cm vs 0cm**	2.831 [1.157-6.928] 0.023		-	-
**Rt > 2cm vs 0cm**	2.311 [0.994-5.373] 0.052		-	0.152 [0.025-0.904] 0.038

TP regimen				
	Analysis of prognosis		Analysis of prediction	
	OS (64/74)^a^ HR [95% CI] p	DFS (43/49)^a^ HR [95% CI] p	PS (43/74)^a^ OR [95% CI] p	CR (49/74)^a^ OR [95% CI] p
**c.690G>T present vs absent**	NS	NS	NS	NS

PC+TP regimens				
	Analysis of prognosis		Analysis of prediction	
	OS (105/118)^a^ HR [95% CI] p	DFS (70/78)^a^ HR [95% CI] p	PS (65/118)^a^ OR [95% CI] p	CR (78/118)^a^ OR [95% CI] p
**c.690G>T present vs absent**	**1.771 [1.129-2.777] 0.013**	NS	NS	NS
**Type (serous vs non-serous)**	0.581 [0.350-0.963] 0.035			
**FIGO IV vs (IIB+IIC)**	1.924 [1.024-3.615] 0.042			
**Rt ≤ 2cm vs 0cm**	2.683 [1.551-4.641] <0.001			
**Rt > 2cm vs 0cm**	3.300 [1.823-5.974] <0.001			

a Values before and after a slash (/) in the analyses of prognosis stand for the number of completed observations vs all observations, respectively, whereas the same values in prediction tests represent the number of tumors positively responding to the treatment vs all tumors. Only the results with p-values < 0.1 are shown and those with p-values < 0.05 are highlighted in bold type. HR, OR, and CI stand for the hazard ratio, odds ratio, and confidence interval, respectively. OS – overall survival; DFS – disease-free survival; PS – platinum sensitivity; CR – complete remission; NS – a non-significant result (p ≥ 0.1).

In line with the prognostic results, the multivariate analysis of tumor response to chemotherapy revealed associations between the presence of the *CEBPA* c.690 G>T SNP and almost 7-fold decrease of sensitivity to the PC treatment (OR 0.144, 95% CI 0.027-0.778, p=0.024) (Figure 1C). Accordingly, the chance for complete remission was over 5 times lower in patients harboring this polymorphism than in non-carriers, though this result was on the border of statistical significance (OR 0.188, 95% CI 0.033-1.063, p=0.059) (Table 2).

### Expression studies

**Figure 2:**
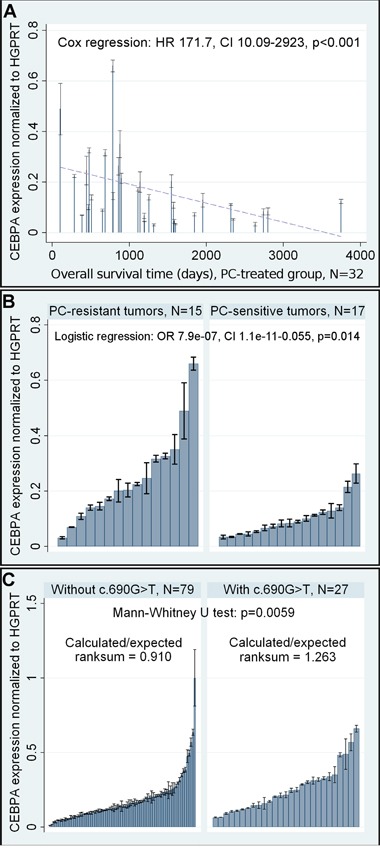
Selected results of the statistical analysis of *CEBPA* mRNA expression **A.** Evaluation of a prognostic value of altered *CEBPA* expression at the mRNA level in the PC-treated group. A dashed linear regression line is shown to visualize the trend of expression. **B.** Evaluation of a predictive value of altered *CEBPA* expression at the mRNA level in the PC-treated group. **C.** The analysis of a relationship between the presence of *CEBPA* c.690G>T SNP and elevated expression of *CEBPA* at the mRNA level. In all graphs, black lines on bars represent standard deviations of RT-qPCR measurements for each tumor.

*CEBPA* gene expression in ovarian cancer patients was evaluated at the RNA level with Reverse Transcription quantitative PCR (RT-qPCR). Noteworthy, although tumors from both the PC- and TP-treated patients were tested with this method, we obtained statistically significant results only for the former group. The multivariate Cox and logistic regression models showed that *CEBPA* overexpression was a negative prognostic and predictive factor in these patients. This unfavorable clinical outcome was seen with all clinical measures assessed, including overall survival (OS), disease-free survival (DFS), and response to chemotherapy (Table 3, Figure 2A-2B).

**Table 3:** The *CEBPA* mRNA expression – statistical results of the multivariate analysis of prognosis (Cox proportional hazards model) and prediction (logistic regression model) in the PC-treated group, TP-treated group, and the joined PC- and TP-treated groups of ovarian cancer patients

PC regimen				
Variable name	Analysis of prognosis		Analysis of prediction	
	OS (31/32)^a^ HR [95% CI] p	DFS (20/22)^a^ HR [95% CI] p	PS (17/32)^a^ OR [95% CI] p	CR (22/32)^a^ OR [95% CI] p
***CEBPA* expression (high vs low)**	**171.7 [10.09-2923] <0.001**	**11728 [19.84->99999] 0.004**	**7.85e-07 [1.12e-11-0.055] 0.014**	**0.0002 [1.32e-07-0.512] 0.033**
**Grade 4 vs (1+2)**	3.413 [1.333-8.739] 0.010	-	-	-
**Rt > 2cm vs 0cm**	2.908 [1.083-7.806] 0.034	-	-	-
**Rt ≤ 2cm vs 0cm**	3.495 [1.206-10.13] 0.021	-	-	-

TP regimen				
	Analysis of prognosis		Analysis of prediction	
	OS (64/74)^a^ HR [95% CI] p	DFS (43/49)^a^ HR [95% CI] p	PS (43/74)^a^ OR [95% CI] p	CR (49/74)^a^ OR [95% CI] p
***CEBPA* expression (high vs low)**	NS	NS	NS	NS

PC+TP regimens				
	Analysis of prognosis		Analysis of prediction	
	OS (95/106)^a^ HR [95% CI] p	DFS (63/71)^a^ HR [95% CI] p	PS (60/106)^a^ OR [95% CI] p	CR (71/106)^a^ OR [95% CI] p
***CEBPA* expression (high vs low)**	NS	NS	NS	NS

a Values before and after a slash (/) in the analyses of prognosis stand for the number of completed observations vs all observations, respectively, whereas the same values in prediction tests represent the number of tumors positively responding to the treatment vs all tumors. Only the results with p-values < 0.1 are shown and those with p-values < 0.05 are highlighted in bold type. HR, OR, and CI stand for the hazard ratio, odds ratio, and confidence interval, respectively. OS – overall survival; DFS – disease-free survival; PS – treatment sensitivity; CR – complete remission; NS – a non-significant result (p ≥ 0.1).

We found that the presence of c.690G>T SNP (rs34529039) was related to the elevated expression of *CEBPA* mRNA (Mann-Whitney U test p=0.0059, Figure 2C). This explains the concordance between the results of gene expression and genotyping studies in relation to patient outcome.

No significant correlation was found between levels of *CEBPA* mRNA expression or the presence of the *CEBPA* gene polymorphisms and the histological type of the tumor, tumor grade, tumor stage or patient age.

## DISCUSSION

To date, the *CEBPA* c.690G>T SNP (rs34529039) was analyzed only in acute myeloid leukemia (AML) patients, where it occurred with a frequency ranging from 3.6% to 32%, and was described as a non-pathogenic alteration [11, 12]. Its impact on cancer risk, prediction and prognosis has not yet been evaluated.

This is the first study to investigate the presence and significance of expression and gene status of *CEBPA* in ovarian cancer. For the first time, we suggest here that both the c.690G>T SNP and overexpression of *CEBPA* mRNA are negative prognostic and predictive factors in ovarian cancer patients treated with DNA-damaging agents (the PC regimen). We also showed that the presence of this SNP positively correlated with the elevated expression of *CEBPA* mRNA.

The c.690G>T SNP is a synonymous polymorphism in the Thr230 locus of the CEBPA protein which changes one threonine codon to another. Thus, the protein sequence remains the same. Nevertheless, some recent studies proved that synonymous codon substitutions may affect the kinetics of mRNA translation when frequency distribution of both codons in the genome significantly differs [13]. In compliance with these findings, in most of the human population, the CEBPA Thr230 is encoded by the ACG triplet, which occurs with the frequency equaling 6.1 per 1000 codons (http://www.kazusa.or.jp/codon/). By contrast, the ACT codon, present in the c.690G>T SNP carriers, appears more than twice as often in the human genome (13.1 per 1000 codons). Furthermore, the latter codon is recognizable by more tRNA types than the dominant one due to a phenomenon known as the wobble base pairing. The difference in the prevalence of the aforementioned nucleotide triplets, and likely also the corresponding tRNA molecules [14], may change the rate of ribosome traffic, thus affecting the translation, folding, and subsequent modifications determining the CEBPA activity and function. The miRNA-based regulation of *CEBPA* expression may be impaired by such a codon alteration, too [15]. In accordance, some synonymous SNPs naturally occurring in the genome were shown to contribute to the development of various neoplasms, like AML, melanoma, and other types of cancer [15, 16].

Noteworthy, the Thr230 residue of CEBPA is known to be phosphorylated by the glycogen synthase kinase (GSK3). This mechanism plays a crucial role in the regulation of activity and intracellular levels of the CEBPA protein [17–19]. It is theoretically possible that the “silent” c.690G>T polymorphism at the Thr230 locus may hamper phosphorylation of CEBPA, leading to quantitative and functional aberrations within the CEBPA protein and resultant impairment of its tumor suppressor role.

Herein, *CEBPA* overexpression was significantly correlated with poor prognosis and prediction of ovarian cancer patients, likely due to an oncogenic role that CEBPA apparently acquires in this neoplasm. The literature does not provide an explicit answer, whether CEBPA is an oncogene or a tumor suppressor. On one hand, inactivating mutations in the *CEBPA* gene have been described in about 10% - 15% of AML cases [20] (and rarely in non-hematologic malignancies [20, 21]), and they may result in loss of the protein function. In this disease, CEBPA activity may also be altered by the formation of AML1-ETO and AML1-MDS1-EVI1 fusion proteins, which down-regulates CEBPA expression at either the transcriptional or the translational level, respectively [22, 23]. As for solid tumors, CEBPA protein expression was reported to be markedly diminished in lung, skin and breast cancers [24–26]. Down-regulation of CEBPA due to its promoter methylation was also reported in head and neck squamous cell carcinoma (HNSCC) [27].

On the other hand, CEBPA may also act as an oncogene in AML, since it was shown to inhibit both the intrinsic and extrinsic pathways of apoptosis by epigenetically activating the expression of two antiapoptotic genes, *bcl2* and *FLIP*, respectively [28]. Chapiro et al. [29] reported that up-regulation of CEBPA may lead to the development of precursor B-cell acute lymphoblastic leukemia (BP-ALL). In this malignancy, CEBPA becomes overactive by juxtaposition to the immunoglobulin gene enhancer (the t(14;19)(q32;q13) chromosomal translocation), resulting in overexpression of CEBPA at both mRNA and protein levels.

Similarly, studies on human hepatocellular carcinomas (HCC) [30] showed up-regulation of CEBPA at both mRNA and protein levels. A forced down-regulation of this gene led to reduced colony formation and cell growth. In line with these observations, Yin at al. have shown in their research on prostate cancer that up-regulation of CEBPA may cause the inactivation of the G1/S checkpoint, stimulation of a transition from the G1 to S and G2 phases, stimulation of cell proliferation and enhancement of the anchorage-independent formation of colonies [31]. Inactivation of the G1/S checkpoint may protect cancer cells from apoptosis triggered off by DNA-damaging agents, causing a “replication by-pass”, and potentially worsening the clinical outcome. In recent years, Zhao et al. [32] proved that overexpression of the CEBPA protein inhibits apoptosis elicited by DNA-damaging agents in leukemic cells.

Our study corroborates these data, since all clinical associations of *CEBPA* overexpression and/or the presence of the c.690G>T SNP we found, were confined to the patients treated solely with DNA-damaging agents. None of these associations were observed in the TP-treated patients, who were administered mainly with taxol, a compound with a different mechanism of action, despite the larger size of the latter group. Consistently, the statistical results (related to overall survival only), that we obtained on combining the PC- and TP-treated groups, were also significant, although at a lower level of significance. This supported our preliminary assumption that such analyses ought to be conducted in uniformly treated groups of patients.

Our study throws some light on the mechanisms by which taxanes overcome resistance observed for regimens based on DNA-damaging agents only. Apparently, the *CEBPA* overexpression and/or the presence of the c.690G>T SNP does not interfere with actions of taxanes, which are known to stabilize microtubules, causing the cell cycle arrest in the G2/M phase [33].

DNA-damaging agents only may be used as second- and further lines of chemotherapy for ovarian cancer patients. The clinical associations that we report herein are conceivably potentially applicable to other malignancies treated with DNA damaging agents, such as lung cancers, testicular cancers, melanomas, myelomas and lymphomas [34].

Finally, Yoon and Smart [35] identified nine potential TP53 binding sites in the CEBPA promoter, and demonstrated that CEBPA is a DNA damage-inducible TP53-regulated mediator of the G1/S checkpoint in keratinocytes. Thus, abnormalities within TP53 (which are common in ovarian cancer [36]) may disturb the normal physiological function of CEBPA and change the way it affects ovarian cancer development and prognosis. As a confirmation of this hypothesis, Seipel et al. [37] have recently shown that restoring the TP53 function after treatment with cytotoxic chemotherapy compounds and TP53 restoring non-genotoxic agents induced CEBPA gene expression, myeloid differentiation, and cell-cycle arrest in AML cells. Furthermore, the development of ovarian cancer is considered to potentially depend on the levels of androgens and gonadotropins [38, 39]. Fan et al. [40] proved that the CEBPA and CEBPB proteins are essential for some gonadotropins-dependent processes, such as ovulation, luteinization and terminal differentiation of granulosa cells. Thus, involvement of CEBPA in ovarian carcinogenesis through the pathways associated with hormones cannot be excluded, either.

In summary, we show here for the first time that the c.690G>T SNP (rs34529039) in the *CEBPA* gene, along with *CEBPA* overexpression at the mRNA level, constitute factors of poor prediction and prognosis in ovarian cancer patients treated with DNA-damaging agents. These findings may have implications for the choice of chemotherapy in ovarian cancer patients, and also in patients with other cancers treated with DNA-damaging agents.

## MATERIALS AND METHODS

### Patients and tumors

The studied material comprised ovarian carcinoma tumor samples and blood samples from 118 patients (age range: 20-76 years, median age: 53 years). Samples were collected in the Institute of Oncology, and in the Brodnowski Hospital, Warsaw, Poland in the period between 1995-2010. Medical records were critically reviewed and relevant data extracted by at least two clinicians for patient selection according to the following selection criteria: no chemotherapy before staging laparotomy, adequate staging procedure, International Federation of Gynecologists and Obstetricians (FIGO) stage IIB to IV disease [41], tumor tissue from the first laparotomy available, moderate or poor tumor differentiation, availability of clinical information, including the residual tumor size and follow-up data. All tumors were uniformly reviewed histopathologically, classified according to the World Health Organization [42], and graded in a four-grade scale, in compliance with the standards given by Barber et al. [43].

The clinical material comprised tumor samples from 44 patients treated with cisplatin and cyclophosphamide (the PC regimen; age range: 34-76 years, median age: 55.5 years), and 74 patients treated with cisplatin or carboplatin and taxanes (the TP regimen; age range: 20-74 years, median age: 53 years). We managed to extract RNA of sufficient quality for a Reverse Transcription quantitative PCR (RT-qPCR) analysis of *CEBPA* expression from 106 of 118 tumors. Detailed clinicopathological characteristics of the patients are shown in Table 4, while Table 1 presents numerical characteristics of the analyzed groups.

**Table 4:** A clinicopathological characteristics of patients

	PC regimen (N=44)	TP regimen (N=74)	PC+TP regiment (N=118)
**Age (years)**			
Range (median)	34-76 (55.5)	20-74 (53)	20-76 (53)
**Histological type**			
Serous	38 (86.4%)	58 (78.4%)	96 (81.4%)
Endometrioid	2 (4.6%)	2 (2.7%)	4 (3.4%)
Undifferentiated	1 (2.3%)	7 (9.5%)	8 (6.8%)
Other types	3 (6.8%)	7 (9.5%)	10 (8.5%)
**Histological grade**			
G2	4 (9.1%)	8(10.8%)	12 (10.2%)
G3	25 (56.8%)	44 (59.5%)	69 (58.5%)
G4	15 (34.1%)	22 (29.7%)	37 (31.4%)
**Clinical stage (FIGO)**			
IIB, IIC	2 (4.6%)	2 (2.7%)	4 (3.4%)
IIIA, IIIB	7 (15.9%)	8 (10.8%)	15 (12.7%)
IIIC	30 (68.2%)	57 (77.0%)	87 (73.7%)
IV	5 (11.4%)	7 (9.5%)	12 (10.2%)
**Residual tumor size**			
0 cm	10 (22.73%)	15 (20.3%)	25 (21.2%)
≤ 2 cm	14 (31.8%)	42 (56.8%)	56 (47.5%)
> 2 cm	20 (45.5%)	17 (23.0%)	37 (31.4%)
**Overall survival (days)**			
Range (median)	104-3750 (915.5)	296-5630 (1010)	104-5630 (1007)
**Disease-free survival (days)**			
Range (median)	95-2521 (393)	96-2884 (414)	95-2884 (413)
**Outcome**			
NED	1 (2.3%)	8 (10.8%)	9 (7.6%)
AWD	2 (4.6%)	2 (2.7%)	4 (3.4%)
DOD	41 (93.2%)	64 (86.5%)	105 (89.0%)
**Sensitivity to treatment**			
Sensitive	22 (50%)	43 (58.1%)	65 (55.1%)
Resistant	22 (50%)	31 (41.9%)	53 (44.9%)
**Response to therapy**			
Complete remission	29 (65.9%)	49 (66.2%)	78 (66.1%)
Other^a^	15 (34.1%)	25 (33.8%)	40 (33.9%)

NED – no evidence of disease; AWD – alive with disease; DOD – died of disease; NA – not applicable.

a Other responses include: partial remission, progression, and no change.

Response to chemotherapy was evaluated on the basis of patient condition and CA125 3-4 week post chemotherapy. As to the assessment of clinical endpoints, complete remission (CR) was defined as disappearance of all clinical and biochemical symptoms of ovarian cancer evaluated after completion of the first-line chemotherapy and confirmed four weeks later [44]. Tumors were considered sensitive to the treatment when disease-free survival of patients was longer than or equal to six months. Otherwise, tumors were presumed to be resistant [45]. Disease-free survival (DFS) time was assessed only for the patients who achieved complete remission. For the PC-treated group, the follow-up time ranged from 104 to 3750 days (median: 915.5 days); the respective values for the TP-treated group equaled 296 and 5630 days (median: 1010 days). All surviving patients had at least a 2-year follow-up duration. Shorter follow-up times were due to earlier patient death. Completed observations were defined as those where the follow-up ended with patient death (OS) or relapse of a tumor (DFS).

Initially, the control group comprised blood samples from 127 healthy women (age range: 19-75 years, median age: 52 years). Just like ovarian tumors, they were assessed for the presence of polymorphisms and mutations within the *CEBPA* gene using the PCR and Sanger sequencing methods. Later, in order to unequivocally decide the question whether the c.690G>T, p.(Thr230Thr) SNP (rs34529039) impacts on the risk of developing ovarian cancer, the control group was extended to 236 healthy women (age range: 19-75 years, median age: 50 years), and genotyped with Real-Time quantitative PCR (qPCR) (see Table 1).

This study was approved by the Bioethics Committee of Maria Sklodowska-Curie Memorial Cancer Center and Institute of Oncology (ref. no. 39/2007).

### DNA and RNA extraction

Tumors obtained during the surgical procedure as well as the relevant blood samples anticoagulated with EDTA were snap-frozen in liquid nitrogen and stored at -70^°^C. Blood samples from the control group were collected and stored likewise. Tumor cryostat sections were stained with hematoxylin and eosin, and then evaluated by a pathologist (JK) to determine the amount of tumor content and viability. The viable epithelial tumor cell content had to be at least 85%. Genomic DNA was extracted with the use the QIAmp DNA Mini Kit (Qiagen, Hilden, Germany) according to the manufacturer's instructions. RNA was isolated using the RNeasy Plus Mini Kit (Qiagen), equipped with gDNA Eliminator columns. RNA quantity was measured with NanoDrop spectrophotometer (Thermo-Fisher Scientific, Waltham, MA, USA), and its quality assessed on Agilent Bioanalyzer (Agilent Technologies, Santa Clara, CA, USA). RNA integrity numbers (RINs) of the samples ranged from 6.5 to 9.4.

### Molecular analysis of the *CEBPA* gene

Polymerase chain reaction (PCR)-based DNA amplification followed by Sanger sequencing was carried out to identify mutations and polymorphisms within the *CEBPA* gene. The entire coding region of the gene was amplified by PCR in two steps utilizing three pairs of primers, designed with the NCBI Primer-BLAST software, and the *CEBPA* genomic sequence (GenBank Accession No: NG_012022.1). In the first round of PCR, the entire coding sequence (1388 bp) was amplified using two outer primers: (forward, OF) 5^’−^ATGCCGGGAGAACTCTAACT -3^’^ and (reverse, OR) 5′-ACCGGAATCTCCTAGTCCTG- 3^’^. In the second round, two nested PCRs were run using the product of the first PCR reaction as a template, and two pairs of inner primers: (forward, IF1) 5^’−^ATGCCGGGAGAACTCTAACT-3^’^, (reverse, IR1) 5′-CAGGTGCATGGTGGTCTG-3′, and (forward, IF2) 5′-GGCCTCTTCCCTTACCAG-3′, (reverse, IR2) 5′-ACCGGAATCTCCTAGTCCTG-3′. The products of the nested PCR were 685 bp and 668 bp long, respectively). PCR mixtures were prepared according to the standard procedure (Applied Biosystems, Waltham, MA, USA) with addition of 5 mM betaine (Sigma-Aldrich, St. Louis, MO, USA). PCR reactions were performed in the Gene Amp 9700 thermal cycler (Applied Biosystems) with an initial denaturation step at 94^°^C for 10 min, followed by 36 cycles consisting of: denaturation (94^°^C, 90 s), annealing (55^°^C, 45 s), extension (72^°^C, 90 s) in the first PCR, and denaturation (94^°^C for 30 s), annealing (55^°^C, 20 s), extension (72^°^C, 50 s) in nested PCRs. The final extension step (10 min, 72^°^C) was the same in all PCRs.

PCR products were purified enzymatically with exonuclease I and alkaline phosphatase (illustra ExoProStar, GE Healthcare Life Sciences, Little Chalfont, UK), and then sequenced in both directions using the BigDye Terminator v3.1 Cycle Sequencing Kit (Life Technologies, Carlsbad, CA, USA) on an automated ABI PRISM 3100 Sequencer (Life Technologies) according to the manufacturer^’^s recommendations.

Additionally, the c.690G>T, p.(Thr230Thr), (rs34529039) SNP was assessed by qPCR-based genotyping in the extended group of 236 healthy women. This study was carried out on the 7500 Fast Real-Time PCR System (Life Technologies) using two different Custom TaqMan Genotyping Assays (ID: AHCTFCJ, lot numbers: 1500898_B1 and 1505907_A10, Life Technologies), tested by the manufacturer. Only those genotyping calls which were fully consistent for both assays were taken into account. The qPCR reactions were performed in the volume of 11 μl using the SensiFAST™ Lo-ROX Genotyping Kit (Bioline, London, UK) and about 25 ng of genomic DNA per well. The thermal profile of qPCR (the same for both TaqMan assays) was as follows: 60^°^C for 1 min. (pre-PCR read), 95^°^C for 3 min. (hot start of the polymerase) followed by 40 cycles with all the standard ramp speeds decreased by 50%, consisting of three steps: denaturation (95^°^C, 30 s), annealing (59^°^C, 30 s), and extension (60^°^C, 1 min.), and then the final post-PCR read (60^°^C, 1 min.). In each qPCR reaction the ROX dye was used as a passive reference.

### Reverse Transcription quantitative PCR (RT-qPCR)-based studies of *CEBPA* mRNA expression

All RT-qPCRs were performed on the 7500 Fast Real-Time PCR System (Life Technologies), using *HGPRT* as a reference gene. Gene expression was evaluated with TaqMan assays, *CEBPA*-specific (6-FAM-labeled, Life Technologies, assay id: Hs00269972_s1) and *HGPRT*-specific (VIC-labeled, Life Technologies, assay id: 4326321E). RT-qPCRs were run in triplicates as multiplex reactions in the volume of 11 μl using *TaqMan Universal PCR Master* Mix with uracil N-glycosylase (Life Technologies) and about 10-11 ng of total RNA, earlier reverse transcribed to cDNA with the Superscript III First-Strand Synthesis System (Life Technologies). Obtained expression data were quantified using a standard curve prepared from one of the analyzed samples. Efficiency of RT-qPCR reactions ranged from 80% to 110%, as assessed based on slopes of the standard curves. The coefficient of determination (*R*^2^) of the curves was always higher than 0.99 [46].

The reference gene used in this study, *HGPRT*, was nominated from among 11 genes included on TaqMan Human Endogenous Control Plates (Life Technologies), because it was characterized by the most stable expression in both the PC- and TP-treated groups of patients. Expression of the reference genes was assessed for 8 randomly selected tumors from each group. Then, the stability of expression was calculated with the qBase^PLUS^ software (Biogazelle NV, Zwijnaarde, Belgium) [47].

### Statistical analyses

The statistical significance of changes in *CEBPA* mRNA expression and sequence alterations within this gene was assessed using the multivariate Cox proportional hazards model (prognostic value) or multivariate logistic regression model (predictive value). Changes in gene status and levels of expression of *CEBPA* were correlated with clinicopathological tumor characteristics, including: patient age (categorized by median split); residual tumor size; clinical stage (FIGO); histological grade (the last three parameters were categorized as shown in Table 4), and histological type (categorization: serous vs non-serous types). Additionally, the multivariate logistic regression model adjusted for age enabled us to evaluate whether there was a difference in frequency distribution of identified *CEBPA* gene alterations between ovarian cancer patients and healthy women. All multivariate statistical models were simplified by backward stepwise elimination of variables if their individual p-values were higher than or equal to 0.1.

Afterwards, we used the Mann-Whitney U or Kruskal-Wallis test to determine direct associations of *CEBPA* mRNA expression (continuous data) with each variable included in multivariate analyses, and also with polymorphisms identified herein. In case of categorical data, i.e., the *CEBPA* genotyping results (binomial categorization), the same relationships were looked for, but with the use of chi-square or Fisher's exact probability tests, depending on the size of the analyzed groups. In all the tests the statistical significance level (alpha) was set to 0.05.

Noteworthy, in this study, *CEBPA* mRNA expression was always treated as a continuous variable to avoid arbitrary categorization of data, which could potentially lead to falsification of statistical results. A tumor exhibiting the highest expression of the *CEBPA* transcript was used as a calibrator. Thus, all the expression values ranged from 0 to 1. This approach allowed for approximate estimation of the risks based on the hazards ratios (HRs) and odds ratios (ORs) in a similar way as for categorical variables. However, given that only one tumor (calibrator) had the *CEBPA* mRNA expression equaling 1, and none – equaling 0, the real increase or decrease of the risk was always lower than that estimated on the basis of HRs and ORs shown in Table 3.
